# Characteristics of chronic lymphocytic leukemia in Senegal

**DOI:** 10.1186/s12878-016-0051-y

**Published:** 2016-04-23

**Authors:** Abibatou Sall, Awa Oumar Touré, Fatimata Bintou Sall, Moussa Ndour, Seynabou Fall, Abdoulaye Sène, Blaise Félix Faye, Moussa Seck, Macoura Gadji, Tandakha Ndiaye Dièye, Claire Mathiot, Sophie Reynaud, Saliou Diop, Martine Raphaël

**Affiliations:** Hematology, Cheikh Anta Diop University, Dakar, Senegal; Hematology, Aristide Le Dantec Hospital, Dakar, Senegal; Curie Institute, Paris, France; Hematology, University Hospital, Nice, France; University Paris XI, Paris, France

**Keywords:** Chronic lymphocytic leukemia, Clinic, Cytology, Immunophenotype, Cytogenetic abnormalities

## Abstract

**Background:**

Chronic lymphocytic leukemia (CLL) is a mature B-cell neoplasm characterized by the expansion of CD5-positive lymphocytes in peripheral blood. While CLL is the most common type of leukemia in Western populations, the disease is rare in Africans. Hence, clinical and laboratory data and studies of CLL in Sub Saharan populations have been limited. The aims of this study were to analyze the characteristics of senegalese patients with CLL at the time of the diagnosis and to identify the correlation between clinical characteristics (Binet stage) with age, gender, laboratory parameters and chromosomal abnormalities.

**Methods:**

In this study, we investigated the clinical and laboratory characteristics of CLL in Senegal. A total of 40 patients who had been diagnosed with CLL during the period from July 2011 to April 2015 in Senegal were evaluated. Cytology and immunophenotype were performed in all patients to confirm the diagnosis. The prognosis factors such as Binet staging, CD38 and cytogenetic abnormalities were studied. The statistical analysis was performed using STATA version 13 (Stata college station Texas). Each patient signed a free and informed consent form before participating in the study.

**Results:**

The mean age was 61 years ranged from 48 to 85. There were 31 males and only 9 females (sex ratio M : F = 3,44). At diagnosic, 82.5 % of the patients were classified as having advanced Binet stages B or C. The prognosis marker CD38 was positive in 28 patients. Cytogenetic abnormalities studied by FISH were performed in 25 patients, among them, 68 % (17 cases) had at least one cytogenetic abnormality and 28 % had 2 simultaneous cytogenetic abnormalities.

**Conclusion:**

Africans may present with CLL at a younger age and our data suggest that CLL in Senegal may be more aggressive than in Western populations.

## Background

Chronic lymphocytic leukemia (CLL) is the most frequent form of leukemia in Western countries [1, 2]. The median age at diagnosis ranges between 67 and 72 years and males are more likely to develop the disease than females [3, 4]. CLL is characterized by clonal proliferation and accumulation of mature, typically CD5-positive B-cells within the blood, bone marrow, lymph nodes, and spleen [5]. It is a heterogeneous disease which can present as an aggressive and life threatening leukemia or as an indolent form that will not require treatment over decades. Rai et al. (1975) [6] and Binet et al. (1981) [7] staging systems are the standard clinical staging to estimate prognosis of patients. However, both systems fail to indicate the higher risk of progression among patients in early stages of the disease. These clinical staging systems were complemented by prognostic markers based on : serum prognostic factors, immunoglobulin heavy chain variable region (*IGHV*) mutation status, some cytogenetic abnormalities, cell membrane expression of CD38, and intracellular expression of zeta-associated protein-70 (ZAP- 70) [5, 8, 9].

As CLL is a rare disease in Africa [10, 11], clinical and laboratory data and studies in Sub Saharan populations have been limited. In this first study of CLL in Senegal, we have investigated the clinico-biological characteristics of the disease at time of diagnosis.

The first objective of this current study was to analyze the characteristics of senegalese patients with CLL at the time of the diagnosis. The second objective was to identify the correlation between clinical characteristics (Binet stage) with age, gender, laboratory parameters and chromosomal abnormalities.

## Methods

### Patients

In a prospective study, a total of 40 patients diagnosed with CLL between July 2011 to April 2015, in different hospitals in Senegal were evaluated. The diagnosis was based on morphological and immunophenotypical findings according the World Health Organization (WHO) classification (2008) [12]. The patients were not treated at diagnosis and clinical characteristics including age, gender, symptoms and clinical features were provided by referring physicians. All of the patients were classified using the Binet staging system as one of the three groups (A, B or C). The Binet staging system [7] is based on the number of involved areas, as defined by the presence of enlarged lymph nodes of greater than 1 cm in diameter or organomegaly, and on whether there is anemia or thrombocytopenia. Binet stages are defined as follows:

- Stage A : Hemoglobin (Hb) more than 10 g/dL and platelets above 100 x 10^9^/L and to two of the superfical lymph nodes involved.
- Stage B : Hb above 10 g/dl and platelets above 100 x 10^9^/L and organomegaly greater than that defined for Stage A (i.e., three or more areas of nodal or organ enlargement).
- Stage C : All patients who have Hb of less than 10 g/dL and/or a platelet count of less than 100 x 10^9^/L, irrespective of organomegaly.

### Peripheral Blood cells Counts

The peripheral blood cells counts were performed on the Symex XT2000i ^TM^ (Sysmex Diagnostics, Japan) and a blood smear stained by May Grunwald Giemsa was obtained for all patients.

### Immunophenotypical analysis

In each patient, immunophenotype of leukemic cells was performed by flow cytometry, using the FacsCalibur™ flow cytometer (Becton Dickinson, CA, USA). Different panels of antibodies were used to assess the immunophenotype CLL scoring system proposed by Matutes et al. [13]. These monoclonal antibodies were : CD45-APC/CD19-PerCP/kappa-FITC/lambda-PE/CD5-PE/FMC7-FITC/CD22-FITC/CD23-PE/CD10-FITC/CD38-PE/CD11c-PE/CD25-FITC/CD103-FITC. Data were acquired and analyzed with the BD CellQuest Pro software (Becton Dickinson).

### Fluorescence in situ hybridization

Cytogenetic abnormalities were determined by Fluorescence in situ hybridization (FISH) on peripheral blood using Vysis probes (Abbott) according to the manufacturer recommandations. The following abnormalities : del13q14 (D13S319 probe), del 11q22 (ATM probe), del17p13 (TP53 probe) and trisomy 12 (CEP 12 DNA Probe) were tested.

### Ethical considerations

**Fig. 1:**
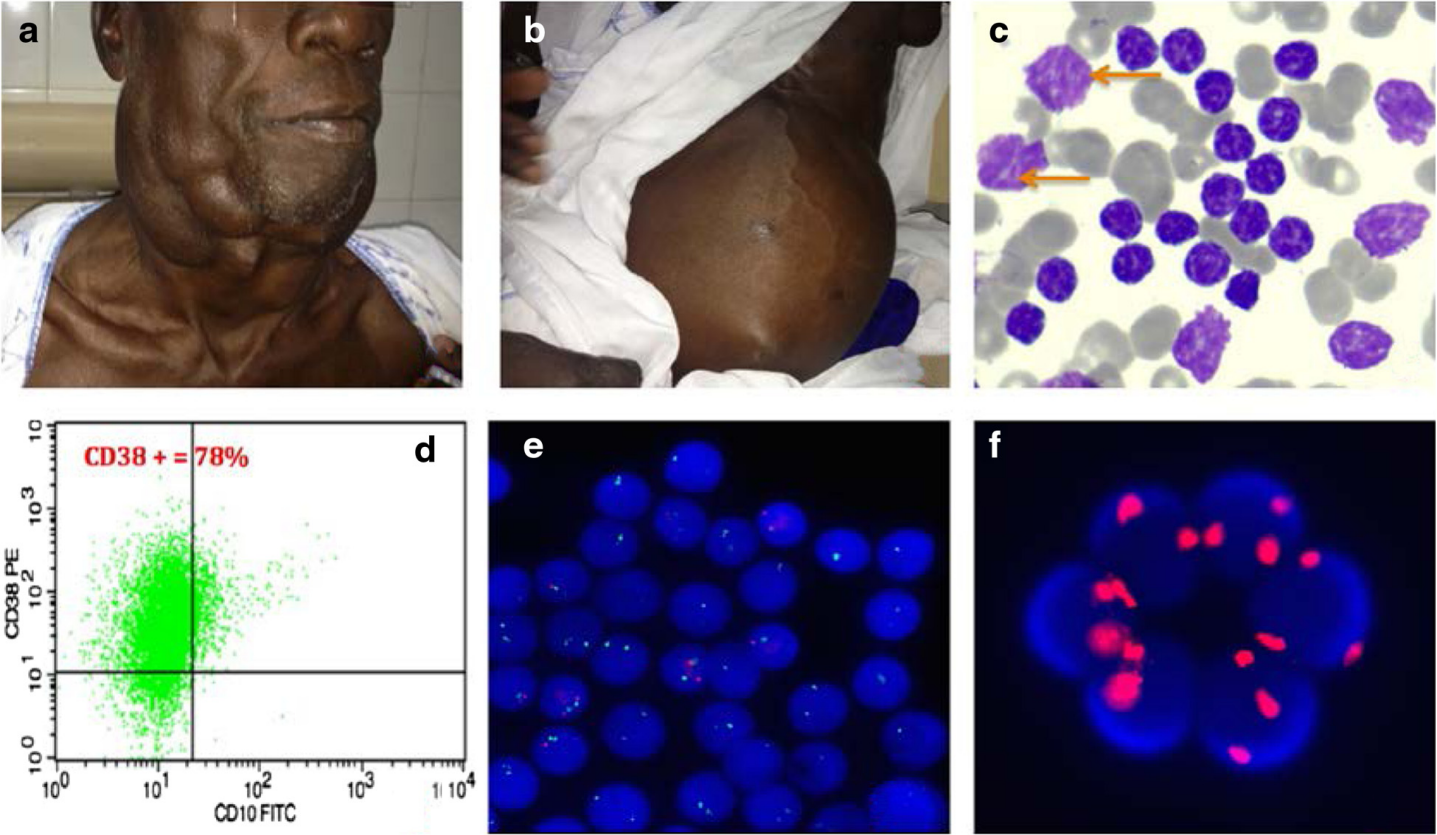
**a** Bulky cervical lymph nodes. **b** Huge splenomegaly with splenic abcess. **c** Peripheral blood smear, typical small lymphocytes,with hypermature clumped chromatin and scanty cytoplasm. Presence of smudge cells (arrows). **d** Immunophenotype with CD38 positivity. **e** FISH, bi allelic deletion of 13q. **f** trisomy 12 (FISH)

The study was approved by the « Research Ethics Committee » of Cheikh Anta Diop University and each patient signed a free and informed consent form before participating. A written consent for publication of personal information, such as that contained in table, was obtained from all participants. Written consent to publish the images contained in Fig. 1 was also obtained from the relevant participant.

### Statistical analysis

Data were collected using Microsoft Excel Spreadsheet and then transferred to Stata for data management and statistical analysis. Continuous variables were described as mean with standard deviation or as median with inter quartile range if the variable was not normally distributed. Qualitative variables were described as proportion. For the bivariate analysis, difference between means was tested using the student *t*-test or the Mann–Whitney non-parametric depending on the normality assumption. Association between categorical variables was performed using the Pearson chi-square test or the Fisher exact test. The estimations were done within a 95 % confidence level and the entire statistical tests were significant when the p value was below the threshold level of 0.05. The statistical analysis was performed using STATA version 13 (Stata college station Texas).

## Results

Of the 40 patients studied, 31 were males and only 9 females (sex ratio M : F = 3,44). The mean age was 61 years ranged from 48 to 85. Table 1 shows the age and sex distribution of the CLL patients in this study. CLL occurred frequently between 55–70 years (55 % of patients). Just over one quarter of patients was under 50 years old.

**Table 1:** Age and sex distribution

Variables	Number	Proportion (%)
Age groups (years)		
<55	11	27.5
55–70	22	55
>70	7	17.5
Sex		
Male	31	77.5
Female	9	22.5

The most frequently found symptoms were related to tumor syndrome; it was mainly discomfort, pain or mass abodominal observed in 80 % of cases. Signs of impaired general condition: weight loss, asthenia or anorexia were found respectively in 52.5- 65 -30 % of cases. There was only one patient with a bleeding symptoms (Table 2).

**Table 2:** Clinical features in our patients

Clinical features	Number (I = 40)	Proportion (%)
Symptoms		
Weight loss	21	52.5
Abdominal pain/discomfort	19	47.5
Abdominal mass	13	32.5
Weakness	26	65
Cervical, axillary or inguinal mass	19	47.5
Anorexia	12	30
Fever	07	17.5
Nght swaet	03	7.5
Bleeding	01	2.5
Signs		
Anaemia- Pallor	25	62.5
Lymphadenopathy (cervical, axillary, inguinal)	36	72
No lymphadenopathy	04	10
Splenomegaly		
- moderate (I, II Hackett)	11	27.5
- gross splenomegaly (III, IV,V Hackett)	16	40
Spleen no palpable	13	32.5
Hepatomegaly	06	15
Respiratory tract infection	04	10

Clinically, tumor syndrome was evident in most patients. The enlarged lymph nodes were present in 72 % of patients (Fig. 1a) and 26 patients had a palpable spleen of which 40 % had at least one splenomegaly according to Hackett type III (Fig. 1b).

The average lymphocytosis was very high : 186.68x10^9^/L, ranges between : 5.03 to 869x10^9^/L. The blood smears showed mature lymphocytes with clumped chromatin and scanty cytoplasm (Fig. 1c). Prolymphocytes, counted in all cases, were present in only 9 cases and the percentage was below 15 %. Smudge cells (Fig. 1c, arrows) were observed in almost all peripheral blood smears except in 3 cases.

The mean hemoglobin level was 9.5 g/dl (Ranges: 3.9 to 15.2), 55 % of patients had an hemoglobin less than 10 g/dl. Thrombocytopenia was observed in 21 patients. The platelets count was below 50x10^9^/L in 6 patients (patients : 3, 15, 18, 23, 32, 40. Table 3) and the mean platelets levels was 149.07x10^9^/L (Ranges: 21- 452x10^9^/L) (Table 3).

**Table 3:** Characteristics of 40 CLL patients

Patients N°	Age/Sex	Lymphocytesx10^9^/L	Haemoglobin g/dl	Platelests x10^9^/L	Binet Stage	CD38 expression*	Deletion of 13q (*n* = 11/25)	Deletion of 11q (*n* = 3/25)	Trisomy 12 (*n* = 7/25)	Deletion of 17p (*n* = 3/25)
1	63/F	33.4	13.3	279	A	+	-	-	-	-
2	50/F	268.8	8.4	140	C	+	-	-	Yes	-
3	65/M	52.248	12.4	24	B	+	Yes	-	-	-
4	53/M	831.14	5.2	69	C	+	Yes	Yes	-	-
5	54/M	225.5	7.5	137	C	Negative	Yes	-	-	-
6	69/M	179.5	4.4	81	C	+	Yes (biallelic)	-	-	-
7	80/M	49	11.2	200	A	Negative	-	-	-	-
8	63/M	230	9.3	206	C	+	-	-	Yes	Yes
9	50/M	58	8.8	66	C	+	-	Yes	-	-
10	51/M	88.55	9.5	97	C	+	-	-	-	-
11	63/M	145.78	9.7	203	C	Negative	Yes	-	-	Yes
12	59/M	42.6	10.3	126	B	+	yes	-	Yes	-
13	67/M	5.8	15.2	220	A	+	-	-	-	-
14	67/F	6.4	11.6	332	A	+	Yes	-	-	-
15	70/M	491.4	6	31	C	+	Yes (biallelic)	-	Yes	-
16	57/F	12.8	13	229	A	+	-	-	-	-
17	80/F	93.34	13.2	157	B	+	Yes	-	Yes	-
18	60/M	82.3	7.9	28	C	Negative	-	-	Yes	-
19	85/M	397	8.9	108	C	+	-	-	-	Yes
20	54/M	87.8	10.7	239	A	Negative	-	-	-	-
21	60/M	16.38	12.6	276	B	+	-	-	-	-
22	63/M	789.2	8	262	C	Negative	-	-	-	-
23	54/M	51.76	4.9	44	C	+	Yes (biallelic)	-	-	-
24	67/M	201.5	10.3	263	B	+	-	-	yes	-
25	60/M	171	12	203	B	+	yes	yes	-	-
26	72/M	15	6.5	115	C	Negative				
27	58/M	7.1	12.1	146	A	Negative				
28	73/M	241	7.6	242	C	+				
29	74/M	12.7	10.9	157	B	Negative				
30	63/M	734	8.8	151	C	Negative				
31	68/M	5.03	12.1	64	C	+				
32	48/M	165.33	6.7	34	C	Negative				
33	60/M	85.5	11	152	B	+				
34	52/M	156.3	11.2	452	B	+				
35	48/F	365.8	9.7	76	C	+				
36	78/F	198	7.4	115	C	+				
37	54/F	27	9.8	88	C	+				
38	59/M	131	12.8	76	C	Negative				
39	64/F	869	9	54	C	+				
40	55/M	114.41	3.9	21	C	+				
Mean	186.68	9.5	149.07							

CD38 expression : positive if >30 %

At diagnosis, 82.5 % of patients dysplayed an advanced Binet clinical stage : 22.5 % were stage B and 62.5 % were stage C. Only 7 patients were in stage A.

The Matutes scoring was 4 or 5 in almost all patients confirming the CLL diagnosis. Three patients had an atypical CLL with a score at 3/5.

The prognosis marker CD38 was positive (Fig. 1d) in 28 patients of the series, including 4 patients in stage A (Table 4).

**Table 4:** Correlation between clinical stage and laboratory findings

Characteristics	Clinical staging system according to Binet and al.		
	Either stage A or B	Stage C	P value*
	N (%)	N (%)	
Age (years) *n* = 40			
<55	2 (12.5)	9(37.5)	0.1
55-70	11 (68.7)	11(45.8)	
<70	3 (18.75)	4 (16.67)	
Sex (*n* = 40)			
Male	12 (75)	19 (20)	0.75
Female	4 (25)	5 (80)	
Average lymphocytes x10^9^/L (*n* = 40)	61.5	269.5	0.005*
At least one cytogenetic abnormality (*n* = 25)			
Yes	06 (24)	11 (44)	0.097
No	06 (24)	02 (08)	
Deletion 13q			
Yes	05 (42)	06 (46.15)	0.82
No	07 (58)	07 (53.85)	
Deletion 11q			
Yes	1 (8.3)	2(15.4)	0.58
No	11 (91.7)	11(84.6)	
Trisomy 12			
Yes	03 (25)	04 (31)	0.74
No	9 (75)	9 (71)	
Deletion 17p			
Yes	00 (00)	03 (24)	0.07
No	12 (100)	10 (76)	
CD38 expression (*n* = 40)			
Positive	12 (75)	16 (67)	0.75
Negative	04 (25)	08 (33)	

Cytogenetic abnormalities studied by FISH were performed in 25 patients, among them, 68 % (17 cases) had at least one cytogenetic abnormality and 28 % had 2 simultaneous cytogenetic abnormalities.

The 13q deletion was found in 44 % of cases (11/25) and 3 patients (patients 6, 15, 23. Table 3) had a biallelic deletion (Fig. 1e). Seven patients had trisomy 12 (Fig. 1f) while 11q and 17p deletions were found in 3 cases each. Note that patients with bi allelic deletion of 13q or 17p deletion were in stage C (Table 3). Twenty four percent of patients on Stage A or B had at least one cytogenetic abnormality versus 66.7 % in stage C. However this difference was not statistically significant (p value = 0.23 Table 4). The deletion of the long arm of the chromosome 11 were observed in 8.3 % of patients with stage A or B and in 15, 4 % of patients classified in the advanced stage (stage C). The statistical test shows that this difference was not significant (p = 0.58). In addition, the deletion of the short arm of the chromosome 17 was not observed in any patient on stage A or B, whereas this abnormality was detected in 24 % of patients on stage C (Table 4).

The number of lymphocytes count was significantly greater in patients with stage C than in those in the group with stages A or B (p = 0.005). The average number of lymphocytes was 269.5 x 10^9^/L in the advanced stage group compared to 61.5 x 10^9^/L in patients classified in stage A or B.

## Discussion

Chronic lymphocytic leukaemia (CLL) is the most common form of leukaemia in Western countries [1, 2] while it is extremely rare in Africa [10, 11]. In 3 years, only 40 patients with CLL were identified in several centers of Senegal with an average age of 61 years (Ranges : 48–85 years). This average age is comparable to Nigerian [10, 11] and Ethiopian [14] studies which found respectively a mean age of 60, 56 and 55 years.

However, this average age at diagnosis is somewhat higher in Western Countries : American (72 years) [5], English (74 years) [15] or French (72 years) [16]. There is at least 10 years between the age of onset of CLL in African compared to Westerners. We speculate that Africans present with CLL at a younger age than Western patients.

These data may support the idea that environmental factors, remaining to be identified, may be involved. It has been postulated that CLL occurring in younger adults in Africa is a consequence of recurrent malaria and other infections, resulting in a polyclonal B-cell proliferation which in an extreme form is hyper reactive malarial splenomegaly [17].

Male dominance has been reported in the most published series [5, 10, 15, 16]. In ours, male dominance was evident with a ratio M/F = 3.44. A different evolution according to gender was however raised and proved [3, 18, 19]. Catovsky et al. [4] demonstrates that CLL runs a more benign clinical course in women than in men. Women were more likely to have Binet stage A than B or C; their overall survival rates at 10 years were better than for men and they had a better overall response to treatment. No good hypothesis have been advanced to explain the observed trend for a better outcome in women. However, the implications of gender differences in the pathogenesis of CLL and its treatment require further studies. Among our 40 cases, 9 were women (4 in stage A or B and 5 in stage C). We have not however found significant differences between men and women compared to Binet stages (p = 0.75).

CD38 is a well-known lymphocyte differentiation antigen with proposed receptor and adhesion molecule functions. In mature circulating B cells, CD38 ligation induced proliferation by promoting the expression of CD25, MHC-II, and certain cytokines [20, 21].

The prognosis role of CD38 in CLL was first proposed on the basis of an immunophenotypical study of CLL cases with known IGHV sequences. CD38 predicted shorter overall survival rates when expressed on 30 % or more CLL cells [22]. Since this report in 1999, CD38 expression has been well established as an independent prognostic factor in CLL by numerous reports, but with various cut-off levels. While Del Poeta et al. [23] and Hamblin et al. [24] proposed 30 % as the best cut-off, others proposed 20 % [25] or even 7 % [26]. Further cooperative studies are still necessary to define a common cut-off level. We use the cut-off of 30 % in our patients. The CD38 were express in 70 % of patients from the series; 12 of them were stage A or B and 16 patients in stage C in the Binet system. We did not find significant difference between the expression of CD38 and the different stages of Binet (p = 0.75).

The others evaluated prognostic factors were cytogenetic abnormalities perfomed by FISH. The 13q deletion was found in 11 patients (44 %), 6 of them were in stage C. Deletions on the long arm of chromosome 13, specifically involving band 13q14 (del (13q14)) represent the single most frequently observed cytogenetic aberration in CLL, occurring in approx. 55 % of all cases [5]. An isolated del 13q14 is typically characterized by a benign course of the disease. Three of our patients had a biallelic deletion of 13q and they were all in Binet stage C. Nevertheless it has been demonstrated that, there was no difference in the baseline characteristics between patients with CLL who had monoallelic or biallelic deletion of 13q. In addition, there was no significant difference in endpoints, including time to treatment [27]. Interestingly, it has been shown that the size of the 13q deletion is associated with outcome, since patients with CLL with larger aberrations have a shorter time to treatment and overall survival, indicating that several genes included in the deletion have an effect on the disease course [28, 29].

Trisomy 12 is detected in 11–16 % of patients at diagnosis [9] and is associated with an intermediate prognosis [9, 30, 31]. Seven of our patients had trisomy 12 (28 %) including 4 stage C Binet (p = 0.74). The genes involved in the pathogenesis of CLL carrying a trisomy 12 are largely unknown. Furthermore, the prognostic relevance of trisomy 12 remains a matter of debate [32].

The deletions of 11q22-q23 and 17p13 are known to be associated with poor prognosis in CLL [5, 9, 31, 32]. The deletion of 11q is most often monoallelic and carried by 10–17 % of patients with CLL [9, 30]. The minimal deleted region is known to encode several tumor suppressor genes including *ATM* which plays an important role in cell cycle regulation. The deletion of 17p is detected at a frequency of 3–7 % at diagnosis [9, 30]. The 17p deletion often involves the entire p-arm, but some losses are focused to the 17p13.1 region, which encodes the *TP53* gene among several other genes. This gene is a key regulator of the cell cycle. The 11q deletion was found in 2 patients in stage C and 1 B stage while the 3 patients with 17p deletion were all in stage C. No significance was found between these poor prognosis deletions and Binet clinical stages (Table 4). This could be explained by the small size of our series as Lai et al. [33] obtained significant differences in the distribution of p53 deletion according to Binet classification system (P = 0.008).

The number of lymphocytes count was significantly greater in patients with stage C than in those in the group with stages A or B (p = 0.005). A high lymphocytosis could be associated to poor prognosis in African with CLL. However Shvidel et al. [34] demonstrated that although CLL patients presenting with hyperleukocytosis at diagnosis generally have an aggressive clinical course, this is not an independent predictor of survival in CLL. In any case, further studies are needed to better define the role of lymphocytosis in prognostic factors for CLL.

## Conclusion

This study helps to define the characteristics of CLL in sub-Saharan Africa. The patient type would be aged 60 years with a major tumor syndrome, higher lymphocytosis to 150 x 10^9^/L, stage C according to Binet clinical stage, positivity of CD38 and at least one cytogenetic abnormality at biological level.

CLL is certainly much less common in Africa than in Western countries but African patients seem to have a worse prognosis compared to Westerners. We assess time to treatment and the time of overall survival at 5 years to better answer this question.

## Abbreviations

CLL: Chronic lymphocytic leukemia
FISH: Fluorescence in situ hybridization
IGVH: Immunoglobulin heavy chain variable region
Zap 70: Zeta-associated protein 70
WHO: World Health Organization
Hb: Hemoglobin
MCH II: Major histocompatibility complex class II
